# nanite: using machine learning to assess the quality of atomic force microscopy-enabled nano-indentation data

**DOI:** 10.1186/s12859-019-3010-3

**Published:** 2019-09-10

**Authors:** Paul Müller, Shada Abuhattum, Stephanie Möllmert, Elke Ulbricht, Anna V. Taubenberger, Jochen Guck

**Affiliations:** 1 Biotechnology Center, Center for Molecular and Cellular Bioengineering, Technische Universität Dresden, Tatzberg 47/49, Dresden, 01307 Germany; 2 JPK BioAFM Center, Colditzstr. 34-36, Berlin, 012099 Germany; 3 Max Planck Institute for the Science of Light, Staudtstr. 2, Erlangen, 91058 Germany

**Keywords:** Machine learning, Atomic force microscopy, Elasticity, Sorting

## Abstract

**Background:**

Atomic force microscopy (AFM) allows the mechanical characterization of single cells and live tissue by quantifying force-distance (FD) data in nano-indentation experiments. One of the main problems when dealing with biological tissue is the fact that the measured FD curves can be disturbed. These disturbances are caused, for instance, by passive cell movement, adhesive forces between the AFM probe and the cell, or insufficient attachment of the tissue to the supporting cover slide. In practice, the resulting artifacts are easily spotted by an experimenter who then manually sorts out curves before proceeding with data evaluation. However, this manual sorting step becomes increasingly cumbersome for studies that involve numerous measurements or for quantitative imaging based on FD maps.

**Results:**

We introduce the Python package *nanite*, which automates all basic aspects of FD data analysis, including data import, tip-sample separation, base line correction, contact point retrieval, and model fitting. In addition, *nanite* enables the automation of the sorting step using supervised learning. This learning approach relates subjective ratings to predefined features extracted from FD curves. For ratings ranging from 0 to 10, our approach achieves a mean squared error below 1.0 rating points and a classification accuracy between good and poor curves that is above 87%. We showcase our approach by quantifying Young’s moduli of the zebrafish spinal cord at different classification thresholds and by introducing data quality as a new dimension for quantitative AFM image analysis.

**Conclusion:**

The addition of quality-based sorting using supervised learning enables a fully automated and reproducible FD data analysis pipeline for biological samples in AFM.

## Background

The mechanical properties of cells and tissues are an important regulator in development, homeostasis, and disease [1–4]. To assess the mechanical properties of tissues at the single cell level, atomic force microscopy (AFM) has emerged as one of the most popular techniques, as it enables the detection of forces over a wide range (5 pN to 100 nN) at a high spatial resolution (down to 10 nm) [5].

In practice, the mechanical characterization of cells and tissues is realized by bringing the AFM cantilever into contact with the sample and recording the force while indenting the sample. In addition to basic indentation experiments, dynamic modes, such as time-dependent stress relaxation, creep compliance, or oscillatory probing [6–11], have been used to assess the viscoelastic properties of cells and tissues. Here, we focus on basic indentation which employs AFM tips of various shapes (e.g. spherical, conical, pyramidal) to indent the sample up to a predefined force (several nN) while recording the force-distance (FD) curve. These FD curves are then preprocessed (tip-sample separation, base line correction, contact point retrieval) and fitted with contact models for the given indenter geometry. For large data sets, preprocessing and fitting is time-consuming and needs specialized personnel. Thus, Minelli et al. proposed machine-learning with a neural network approach, bypassing FD data analysis, to obtain a diagnostic response directly [12]. Though this approach has the advantage of working autonomously, it does not yield quantitative values for the Young’s modulus. To derive a value for the Young’s modulus, the FD data is commonly fitted with the Hertz model, which assumes that the sample behaves like an isotropic and linear elastic solid [13–15]. Since most biological specimens display viscoelastic properties, the Young’s modulus obtained with the Hertz model is often referred to as “apparent Young’s modulus”. With acquisition rates of approximately five curves per minute for biological tissue, 2D FD grids can be recorded, yielding quantitative maps for various parameters such as the apparent Young’s modulus, the maximum indentation depth, or the axial position of the contact point between AFM tip and sample.

Since AFM measurements can be conducted in physiological buffers and at a controlled temperature range, live biological specimens can be probed at near-physiological conditions. Live tissues can be sectioned for analysis, e.g. microtome sections of embedded tissue [16], or measured directly, e.g. biopsy material [17]. In both cases, the preparation of the tissue can be challenging and requires optimization to obtain a flat surface for probing. For instance, the cutting procedure often yields uneven surfaces. In addition, damaged tissue (cell debris and fat) can disturb the cantilever movement. The resulting artifacts distort the FD curve and thus invalidate any model fitted to it. Therefore, curves exhibiting such artifacts are excluded from subsequent analysis steps.

**Fig. 1:**
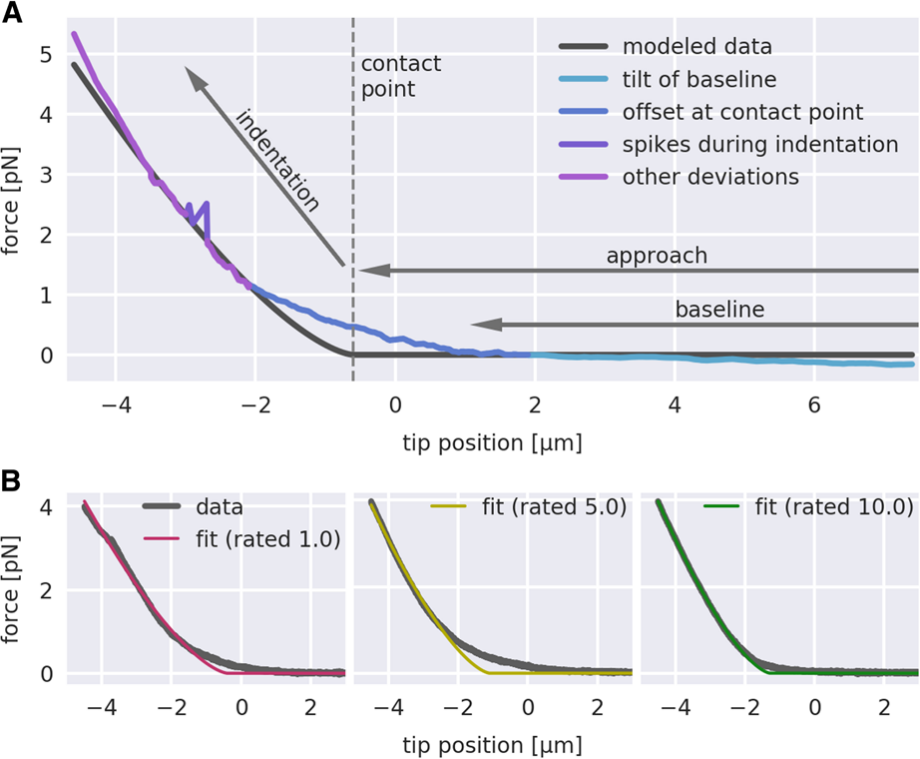
Rating of force-distance (FD) curves. **a** Visualization of several features that degrade the quality of FD curves. **b** Three FD curves with corresponding fits according to Eq. 3 are shown. The colors of the fit label the manual rating (1/magenta: poor, 5/yellow: acceptable, 10/green: good)

Figure 1 illustrates several artifacts commonly observed in nano-indentation experiments. An offset at the contact point may be caused by an uneven surface, resulting merely in a partial contact between the AFM tip and the sample. Sudden spikes in the indentation part originate from slippage of or within the sample. A tilt during the approach part can be caused by contingent contact between the AFM tip and a sample which is insufficiently attached to the cover slide. It is not possible to distinguish between good and poor curves by quantifying the goodness of the fit (e.g. *χ*^2^ test, data not shown). Thus, in a post-measurement step, FD curves with artifacts must be removed manually from the subsequent analysis. However, if the number of curves is large as is the case for densely sampled FD maps of tissue sections, this sorting step becomes too time consuming when performed manually.

Here, we present a machine learning approach that enables a fully automated analysis of biological FD data. The underlying Python package *nanite* covers all aspects of FD analysis, including data import, tip-sample separation, base line correction, contact point retrieval, and model fitting. To automate the removal of artifact-afflicted FD curves, *nanite* employs supervised learning, here using manually rated FD curves of live zebrafish spinal cord sections. With *nanite*, all of these functionalities are made available conveniently via a command-line interface. This approach effectively bypasses the manual and time-consuming analysis process and opens up new ways to classify, compare, and visualize large nano-indentation data sets.

## Results

We applied our rating algorithm to two problems, involving AFM FD data from zebrafish spinal cord sections. The first problem focuses on data quality as a selection criterion. The second problem deals with data quality as an additional imaging dimension.

### Sorting by quality

**Fig. 2:**
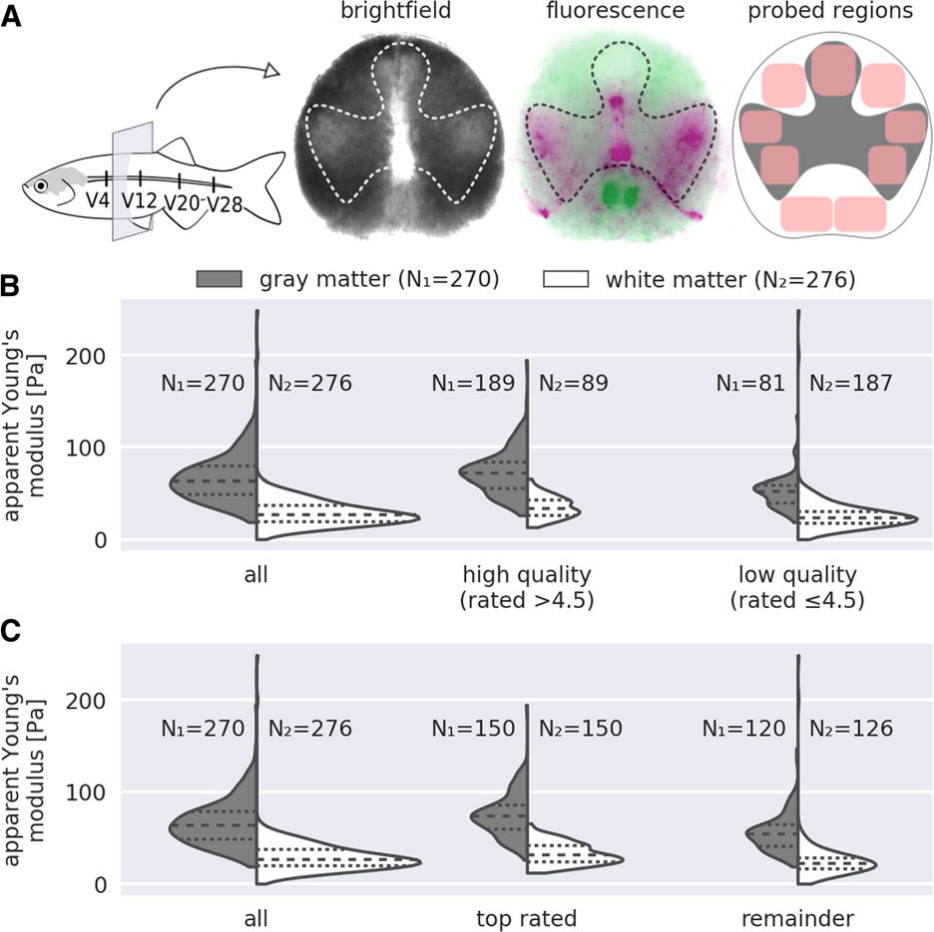
Quality-based sorting. **a** The schematic representation of the adult zebrafish spinal cord indicates the four vertebral levels (V4, V12, V20, and V28) from where tissue sections were obtained. An exemplary tissue section is shown, depicting the locality of gray (inside profile) and white (outside profile) matter. The fluorescence image shows myelin-rich regions labeled with GFP (green, white matter) and mitochondria-rich regions labeled with dsRed (magenta, gray matter). For each probed region (red rectangles), four to five force-distance (FD) curves were recorded. Additional FD curves for white matter were recorded outside of these regions. In total, ten specimens at the age of six months were measured (see [18] for details). **b** The first violin plot shows the distribution of apparent Young’s moduli for gray and white matter. The second and third plot show the same data filtered with the Extra Trees regressor at a classification threshold of 4.5. **c** The first violin plot is identical to that in (B). The second plot shows the 150 top-rated (Extra Trees regressor) data points for gray and white matter. The third plot shows the remainder of the data points. The number of FD curves in each violin plot is indicated with N_1_ for gray matter and N_2_ for white matter

The elastic properties of the zebrafish spinal cord are tissue-dependent. Gray matter exhibits higher stiffness than white matter [18]. Figure 2a illustrates the location of gray and white matter in an exemplary zebrafish spinal cord section, indicating the probed regions for each of the two tissues. We compared a combined dataset from four vertebra sections (V4, V12, V20, and V28) of ten specimens at the age of six months, which has been presented previously (Fig. 3b in [18]). Figure 2b compares the combined dataset to all curves with an Extra Trees rating above and below 4.5. The general trend that gray matter is stiffer than white matter remains. However, there was a positive correlation between apparent Young’s modulus and curve quality which resulted in a preferred selection of gray matter over white matter. The lower rating of the white matter might be related to a higher viscosity that results in a dissipation of energy and thus, worse fits with the Hertz model. To take into account these differences in the selection step, we compared the apparent Young’s moduli of the 150 top rated curves for each tissue type in Fig. 2c. This selection strategy makes it possible to compare gray and white matter with high statistical significance without sacrificing curves due to tissue-dependent quality.

**Fig. 3:**
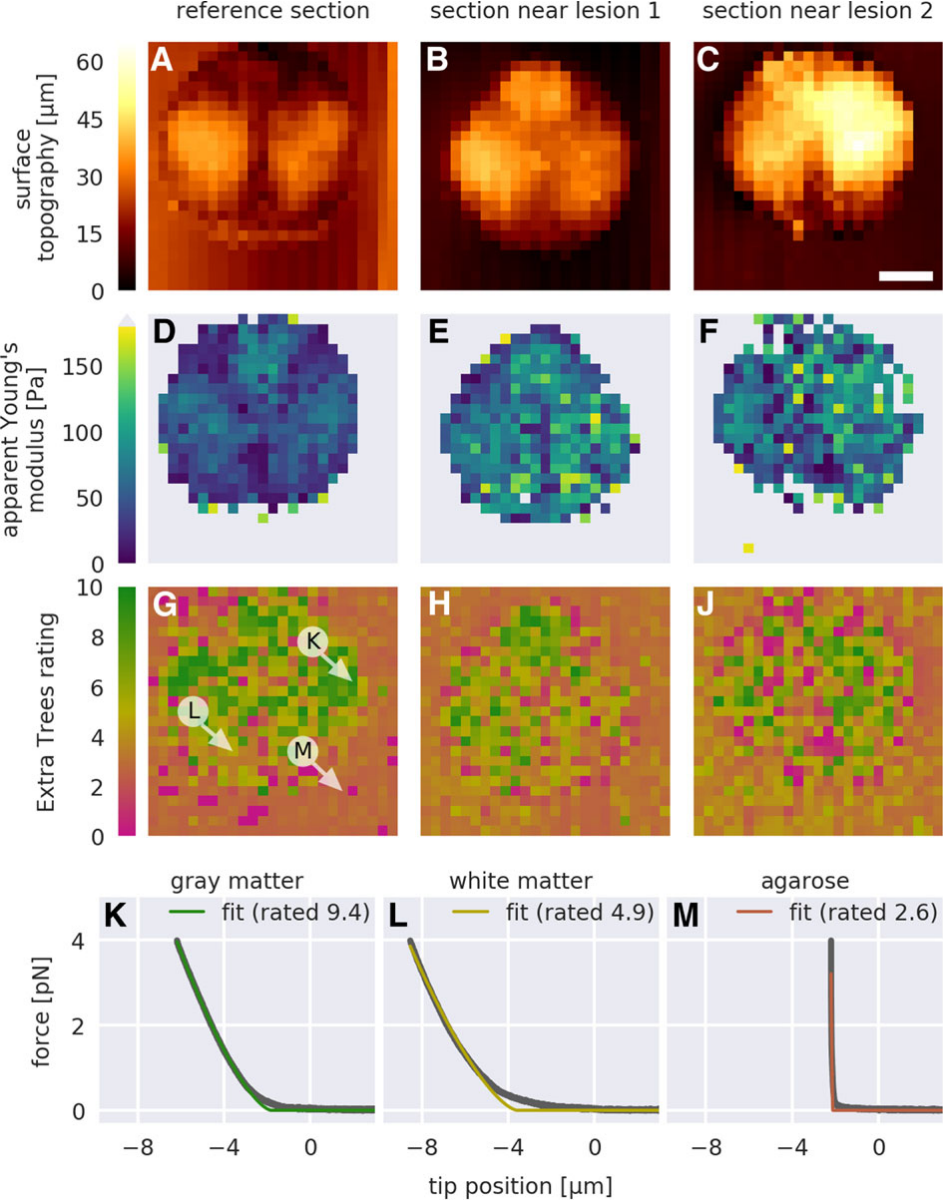
Data quality mapping. For a reference force-distance (FD) map and for two FD maps recorded near lesion sites, **a-c** the normalized minimum height given by the piezoelectric sensor, **d-f** the apparent Young’s modulus obtained with Eq. 3 (gray values are above the color range), and **g-j** the Extra Trees rating are shown. Each pixel represents one FD measurement. Exemplary FD curves and corresponding fits Eq. 3 whose location is indicated with white arrows in (**g**) are shown for **k** the gray matter, **l** the white matter, and **m** the section-embedding agarose. Scale bar in (**c**), 100 **100µ m**

### Visualization of data quality

Quantitative AFM image analysis enables the visualization of regional differences of zebrafish spinal cord sections. Figure 3a-c shows the surface topography images (not tip-sample separation) of the AFM cantilever for three different sections. Gray matter regions appear to protrude from the tissue section (see Fig. 2a for orientation). As discussed in the previous section, the apparent Young’s moduli in gray matter regions were higher than in white matter regions (Fig. 3d-f). In addition, tissue stiffening near lesion sites could be observed (apparent Young’s modulus shown in Fig. 3e, f when compared to Fig. 3d), which has been shown to be correlated to spinal cord repair [18]. The Extra Trees rating visualizes data quality and, in accordance with the findings of the previous section, also correlates with the tissue type (Fig. 3g-j).

**Fig. 4:**
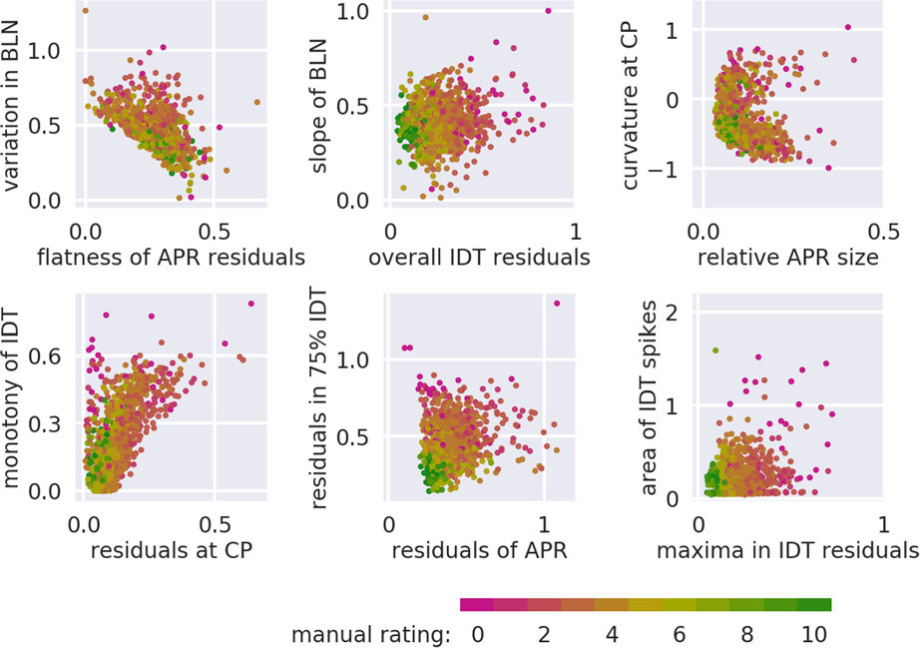
Visualization of the twelve features defined in Table 1. This twelve-dimensional feature space is the training set that we employed for supervised learning. The color of the points indicate the manual rating from magenta (0, poor) to green (10, good)

To give a deeper insight, three exemplary FD curves for gray matter, white matter, and the embedding agarose (indicated in Fig. 3g) are shown in Fig. 3k-m. It should be noted that the low quality attributed to the embedding agarose gel is a consequence of the experimental design and the Extra Trees training step. The indenter was too large to probe the agarose gel with sufficient accuracy and the cantilever was too soft for measuring the large stiffness of the agarose gel. As a result, the indentation depth was comparatively short and the corresponding fit exhibited high residuals. Thus, given the features defined in Table 1 and visualized in Fig. 4, the agarose data exhibited incommensurable conditions resulting in a low Extra Trees rating. Clearly, the analyzed data must be of the same nature as the training data.

**Table 1:** Summary of quality-dependent features used for supervised learning

Number	Feature name	Short description
1	Area of IDT spikes	Area of spikes appearing in the indentation part
2	Curvature at CP	Curvature of the force-distance data at the contact point
3	Flatness of APR residuals	Fraction of the positive-gradient residuals in the approach part
4	Maxima in IDT residuals	Sum of the indentation residuals’ maxima in three intervals in-between 25% and 100% relative to the maximum indentation
5	Monotony of IDT	Change of the gradient in the indentation part
6	Overall IDT residuals	Sum of the residuals in the indentation part
7	Relative APR size	Length of the approach part relative to the indentation part
8	Residuals at CP	Mean value of the residuals around the contact point
9	Residuals in 75% IDT	Sum of the residuals in the indentation part in-between 25% and 100% relative to the maximum indentation
10	Residuals of APR	Absolute sum of the residuals in the approach part
11	Slope of BLN	Slope obtained from a linear least-squares fit to the baseline
12	Variation in BLN	Comparison of the forces at the beginning and at the end of the baseline

The features are visualized in Fig. 4; Abbreviations in feature names: indentation (IDT), contact point (CP), approach (APR), baseline (BLN)

## Discussion

### Performance

The performance of our approach is defined by the choice of the features shown in Table 1, the choice of the regressor (e.g. Extra Trees regressor), and the size of the training set. Most importantly, it is possible to quantify the performance of the regressor as a function of the training set size (see “Methods” section for details). Our approach achieved an average MSE of less than 1.0 rating points and a binary classification accuracy above 87%, which is sufficiently accurate to visualize AFM data quality and to facilitate quality-based sorting.

**Fig. 5:**
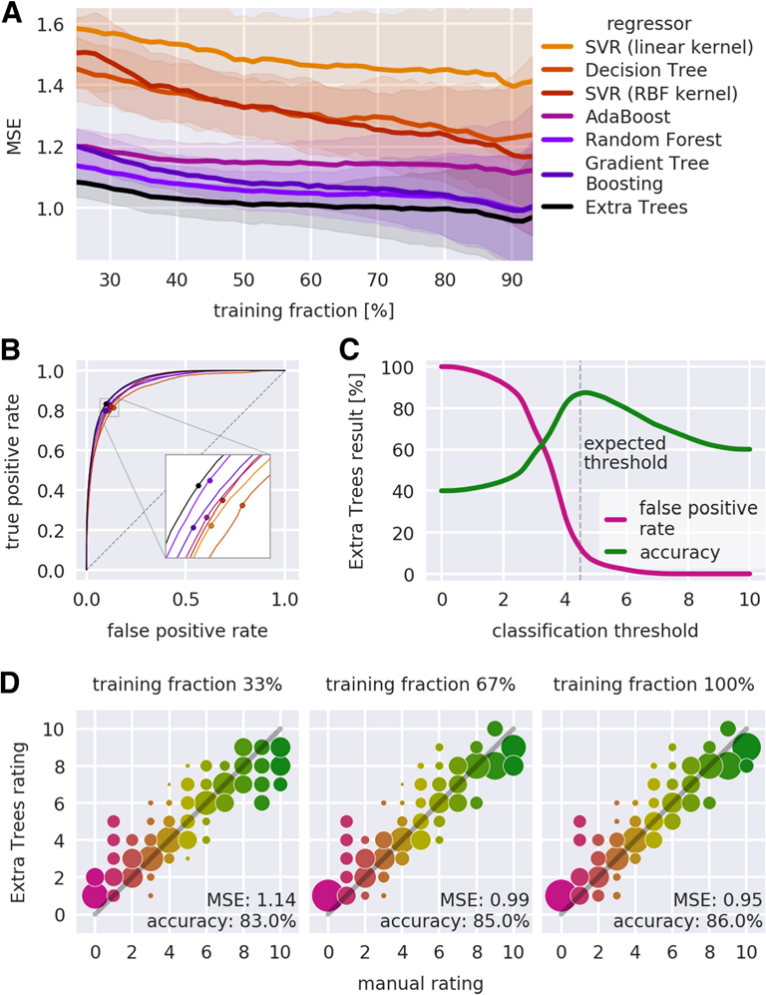
Quantification of regressor performance. **a** Mean squared error (MSE) in dependence of the training fraction. The training fraction is shown in percent of the total number of samples (*N* = 1132); the testing fraction consists of the remaining samples. For all regressors (color code), the average (solid lines) and the standard deviation (shaded regions) were computed from 100 repetitions, each with a different random split into training and testing fraction. The regressors are described in the main text. **b** Average receiver operating characteristics (ROC) graphs. For all regressors (same color code), an average was computed from 100 ROC graphs, each computed using a random split of the samples into two equal parts for training and testing. The points indicate the position of highest accuracy (zoom in inset). The dashed diagonal line indicates the ROC graph of a random classifier. **c** Accuracy and false positive rate for the classification with the Extra Trees regressor. The expected classification threshold at a rating of 4.5 (vertical line), which was defined in the manual rating process, is close to the maximum of the classification accuracy. **d** Visualization of the Extra Trees performance in dependence of the training set size. The training set was randomly split into a testing fraction of 200 samples and a training fraction. From the training fraction, 33%, 67%, or 100% were used for training the Extra Trees regressor which was then applied to the testing fraction with the resulting ratings rounded to integer values. The area of each circle represents the number of samples rated with the Extra Trees regressor normalized to the number of curves per manual rating. Colors represent the manual rating. The MSE and the ROC classification accuracy (threshold at 4.5) are shown in the bottom right corner of each plot. The gray-shaded line indicates a slope of one

Sorting FD curves according to data quality allows the exclusion of unusable data from a subsequent analysis. In principle, two thresholding strategies could be applied (see “Regressor selection” section): maximizing the accuracy (classification threshold at 4.5 in Fig. 5c) or reducing the false positive rate (classification threshold at 6 in Fig. 5c). While the former strategy maximizes the number of curves in subsequent analysis steps, the latter strategy ensures that only a very small percentage of poor FD curves (here 2.1%) is used in the final analysis. Thus, it is possible to dynamically balance quality and quantity in the sorting step.

### Consistency

In order for our approach to work, the features extracted from the analyzed data and those extracted from the training data must represent data quality in similar ways. This can be achieved by enforcing the same measurement protocol (setup used, sample preparation, measurement conditions) and by using the same type of tissue for training and analysis. For instance, different AFM setups might exhibit different levels of background noise or differing readout frequencies. An extreme case of mismatch between training and analysis data is shown in Fig. 3m for agarose, which is discussed in “Visualization of data quality” section. Thus, the performance of a regressor that is trained with data from one setup but applied to data from another setup could be impaired due to feature inconsistency.

### Outlook

There are multiple more or less obvious ways to enhance data analysis and improve compatibility with existing analysis pipelines. For instance, there might be other regressors than those discussed here that could achieve even higher accuracies. In addition, for other experimental data, different hyper-parameters might yield better results than those presently defined in *nanite*. In principle, it could be possible to achieve higher accuracies by increasing the training set size (here *N*=1132). However, a more promising approach would be to improve the quality of the training set. For instance, the integer-valued manual ratings could be mapped from a discrete to a continuous space via an additional comparison of FD curves within the training set. At the same time, new features could be found that allow a better characterization of FD curves. The future development of *nanite* will also include the implementation of existing models for additional tip geometries or for extended models that include, for instance, the contribution of adhesion work. This will allow to better capture the data quality of biological FD data.

## Conclusions

We have demonstrated a novel method that automates the assessment of AFM FD data quality for biological tissues. Our machine learning approach introduces data quality as a new dimension for quantitative AFM image analysis and allows the automated sorting of measurements according to quality. The automation of loading, fitting, and rating experimental data, as provided by *nanite*, heavily speeds up current analysis pipelines which are mostly based on proprietary software and on software that does not by itself take into account data quality during analysis. For the average FD curve, preprocessing and fitting typically takes less than 40 ms while computing the features and rating takes approximately 250 ms on a modern computer. Thus, the proposed rating method could in principle be employed in real-time applications. In addition, the tunable discrimination between good and poor FD data has the potential to greatly simplify prevalent data analysis procedures. For instance, this would allow to map biopsies in an automated manner over a large area for diagnostic purposes, not requiring the presence of highly specialized personnel. In addition, it should be noted that our approach is not limited to the analysis of tissues, but can be applied to other problems, e.g. the measurement of many cells, in the same way. To our knowledge, *nanite* is the first software that quantifies the quality of FD data. This achievement is seminal for the biomechanical characterization of cells and tissues, as it enables the implementation of reproducible analysis pipelines from raw data to data evaluation without manual intervention.

## Methods

Our approach can be summarized as follows. Experimental FD curves are fitted with an appropriate model function and are manually rated. In addition, a set of predefined features is extracted for each curve automatically. Together, these features and the manual ratings form the training set. A regressor that is trained with this training set is then used to predict the ratings of other curves based on their features.

### Sample preparation

All zebrafish were kept and bred under standard conditions as described in [19]. The transgenic line Tg(mbp:GFP) was established and provided by the laboratories of Cheol-Hee Kim, Chungnam National University, South Korea, and Hae-Chul Park, Korea University Ansan Hospital, South Korea [20]. The transgenic line Tg(alpha1-tubulin:mls-dsRed) was established in the laboratory of Carla Koehler, UCLA, USA and provided by Christopher Antos, CRTD, Germany. All experiments were carried out with Tg(mbp:GFP, alpha1-tubulin:mls-dsRed) fish and wild type fish (wik). All experiments comprise male and female fish. All zebrafish were at least three months old. The data recorded from 23 specimens were used in this study (10 specimens for the training set assembly in “Training set assembly” section, 10 specimens for the sorting analysis in “Sorting by quality” section, and 3 specimens for the visualization of data quality in “Visualization of data quality” section).

All zebrafish were sacrificed by immersion in ethyl 3-aminobenzoate methanesulfonate (MS-222, 0.1% in phosphate buffered saline, Sigma-Aldrich, A5040) until five minutes after the respiratory movement of the opercula stopped. This was followed by subsequent immersion in ice-cold water as recommended in [21]. Sacrificed zebrafish were dissected, embedded in agarose, and sectioned with a vibrating microtome as described in more detail in [18].

As all data presented in this study were reutilized from [18], no additional animals were sacrificed for the present study.

### Nanoindentation measurements

AFM calibration and indentation measurements were performed as described in more detail in [18]. Indentation experiments and simultaneous fluorescence microscopy were conducted with a motorized precision stage (CellHesion200, JPK Instruments, Berlin) and the upright Axio Zoom.V16 stereo microscope with a PlanApo Z 0.5 × objective (Carl Zeiss Microscopy, Jena). The AFM probe consisted of polystyrene beads (d = 37.28 ± 0.34 **µ m**, (d = 37.28 ± 0.34 **µ m**, Microparticles GmbH, PS-F-37.0) glued to tipless silicon cantilevers (Arrow-TL1, were carried out on transverse tissue sections at specific regions of interest that belong to either gray or white matter. To broaden the variety of FD curve quality, multiple sections along the anterior-posterior axis (4th, 8th, and 12th vertebrae), partially subject to spinal cord lesions, were used.

To include the choice of model in the rating process, FD curves were first fitted and then rated. Prior to fitting, the tip position (tip-sample separation) was computed, the tip position was set to zero at an approximated contact point using a baseline analysis, and the measured force was corrected for an offset using the baseline average. For fitting, we employed the Hertz model for a spherical indenter

1) (2 $$\begin{array}{@{}rcl@{}} F &=& \frac{E}{1-\nu^{2}} \left(\frac{R^{2}+a^{2}}{2} \ln \! \left(\frac{R+a}{R-a}\right) -aR \right),\\ && \mathrm{with~~} \delta = \frac{a}{2} \ln \! \left(\frac{R+a}{R-a}\right). \end{array} $$

Here, *F* denotes the indentation force, *E* the apparent Young’s modulus, *ν* = 0.5 the Poisson’s ratio, *R* = 18.64 µm the indenter radius, *a* the radius of the circular contact area between bead and sample, and *δ*=*δ*_*t*_−*δ*_*c*_ the indentation depth with *δ*_*t*_ the tip position and *δ*_*c*_ the contact point [22, 23]. Given that this model does not have a closed-form expression, we approximated it by combining the Hertz model for a parabolic indenter with a polynomial correction factor based on a truncated power series approximation (personal communication, Wolfgang Dobler, JPK Instruments, Berlin):

3 $$\begin{array}{@{}rcl@{}} F &=& \frac{4}{3} \frac{E}{1-\nu^{2}} \sqrt{R} \delta^{3/2} \Bigg[1 - \frac{1}{10} \frac{\delta}{R} - \frac{1}{840} \left(\frac{\delta}{R}\right)^{2} \\ && + \frac{11}{15120} \left(\frac{\delta}{R}\right)^{3} + \frac{1357}{6652800} \left(\frac{\delta}{R}\right)^{4} \Bigg] \end{array} $$

This approximation achieves high accuracy, with errors that are below four orders of magnitude relative to the maximum indentation force (data not shown). To reduce the impact of the (commonly large) fit residuals near the contact point *δ*_*c*_, they were suppressed by multiplication with a linear ramp within the interval (*δ*_*c*_−2 µm,*δ*_*c*_+2 µm), which corresponds to approximately ±10 % of the indenter radius *R*. The parameters *E* and *δ*_*c*_ were varied during fitting. After fitting, the FD curves and fits were manually (subjectively) rated on a scale from 0 (poor) to 10 (good) in discrete steps, where curves rated with 5 were considered just usable.

### Training set assembly

To render a machine learning-based rating algorithm possible, it is crucial to find a measure of quality for individual FD curves. The goodness of fit (e.g. *χ*^2^ test) alone is not sufficient to capture the nature of the distinct artifacts shown in Fig. 1a. Therefore, we designed several features, each of which capturing a different aspect of data quality, e.g. contact point position or trends in the fit residuals, while keeping computational costs at a low level. The selection of features is a critical step, because they must be able to capture the experimenter’s notion of data quality. Features were divided into two classes, binary and continuous. Three binary features were used for preprocessing (see below) and twelve continuous features were used for training (see “Regressor selection” section). To simplify the visualization of the feature space, the features were designed to have a small spread, which was partially achieved by applying a logarithmic filter. A short description of each feature is given in Table 1. All features are extracted automatically and form the so-called sample of an FD curve.

The training set was assembled using the samples and the corresponding manual ratings. We preprocessed the training set by removing unusable curves using the binary features. These binary features identify measurements whose fitted contact point is outside of the data range, whose size (combined approach and indentation) is less than 600 data points, or whose indentation part exhibits more than five distinct spikes (see e.g. Fig. 1a). In addition, FD curves for which a feature could not be computed were removed from the training set. For this study, we manually rated 1132 FD curves from zebrafish spinal cord sections. To assure that the training set exhibited a broad quality range, we used a heterogeneous set of samples (different vertebral levels, healthy and scarred tissue, gray and white matter). The resulting training set, visualized in Fig. 4, gives a brief insight into which feature combinations could be relevant for defining the global quality of an FD curve.

### Regressor selection

To connect the features in the training set to the corresponding manual rating, we used a supervised learning approach. Supervised learning utilizes the connection between the computed features and the manual ratings in the training step. Predicting the quality of FD curves based on predefined features is a regression problem. Since each feature captures a different aspect of data quality and, thus, a particular rating may encompass a complex interplay of features, we put our main focus on regressors based on decision trees.

For the present study, we made extensive use of the Python library scikit-learn [24] which comes with a comprehensive set of regressors and associated tools for supervised learning. Their working principles are not discussed here for brevity reasons. The training set was weighted according to the occurrence of ratings. Depending on which regressor was used, we applied an additional preprocessing step to the training set. For the support vector machine regressors (SVR), a linear discriminant analysis was applied and the training set was scaled such that the features were centered at zero with a variance that was comparable for all features. The hyper-parameters of each regressor were determined using an extensive grid search. Thereby, we obtained a set of regressors of which each was optimized for the given training set.

An overview of the performance of all regressors is shown in Fig. 5a. The training set was randomly split into training fraction (used to train the regressor) and testing fraction (used to test the prediction of the regressor) at different percentages. For each percentage, this process was repeated 100 times and the average mean squared error (MSE) was compared. The average MSE quantifies by how many rating points on average a prediction deviates from the manual rating. The basic Decision Tree regressor and the linear SVR performed worst, indicating either overfitting or lack of complexity to address the regression problem. Ensemble methods such as AdaBoost, Random Forest, Gradient Tree Boosting, and Extra Trees better captured the rating process. The Extra Trees regressor yielded the best results, with an average MSE reaching values below 1.0. Thus, the Extra Trees regressor was used in the present study.

Figure 5b shows the receiver operating characteristics (ROC) graphs for all regressors. ROC graphs visualize the performance for classification problems [25], plotting the true positive rate

4 $$ \text{tp\ rate} = \frac{\text{positives\ correctly\ classified}}{\text{total\ positives}}  $$

versus the false positive rate

5 $$ \text{fp\ rate} = \frac{\text{negatives\ incorrectly\ classified}}{\text{total\ negatives}}.  $$

The ROC graph of a random classifier corresponds to the diagonal (0,0) → (1,1) (dashed line in Fig. 5b). A perfect classifier would follow the path (0,0) →(0,1) → (1,1). Thus, the further an ROC curve extends towards the upper left in ROC space, the better its associated classifier. Here, we consider a classification into good (rating above 4.5) and poor (rating below 4.5) data quality. The training set was randomly split into two equal-sized fractions. The first half was used to train the regressor and the second half was used for testing. This process was repeated 100 times with random splits to obtain an average ROC graph. For all regressors, the ROC graphs run along the upper half space above the diagonal, indicating good classification performance. The best classification performance was achieved by the Extra Trees regressor, with an ROC graph closest to the upper left corner (see inset in Fig. 5b). For each of the averaged ROC graphs, the point of maximum accuracy

6 $$ \text{accuracy} = \frac{\text{true\ positives} + \text{true\ negatives}}{\text{total\ testing\ set\ size}}.  $$

is depicted as a point. The Extra Trees classifier achieved the highest accuracy (87.4%) at a classification threshold of 4.64. Thus, the result of the ROC analysis is consistent with that of the MSE analysis described above.

Figure 5c further visualizes the performance of the Extra Trees regressor in the classification problem. At the expected classification threshold of 4.5, the Extra Trees regressor achieved a classification accuracy of 87.1%. The discrepancy between expected (4.5) and actual (4.64) position of the classification threshold is small, considering the fact that the manual ratings are integers. Notably, a threshold of 6 has a false positive rate of only 2.1%, but still achieves a classification accuracy of 79.9%. Thus, FD curves can be sorted into good and poor curves with a tunable specificity.

Figure 5d visualizes the improvement in rating prediction for 200 randomly chosen FD curves when the number of curves used for training is increased. As expected, a larger training fraction reduced the MSE and increased the classification accuracy, improving the prediction performance. In addition, a larger training fraction caused a higher correlation between the Extra Trees rating and the manual rating, as can be seen by an increased alignment of the data points to a slope of 1. Thus, learning-based rating with the Extra Trees regressor in combination with the given training set forms a robust framework that is sufficiently accurate to rate other experimental FD curves, especially, but not limited to, zebrafish spinal cord tissue sections.

## Data Availability

The datasets generated and analyzed during the current study are available on Figshare (https://ndownloader.figshare.com/files/13481393) [26]. The employed method is implemented in the Python package *nanite*, available at https://github.com/AFM-Analysis/nanite. The documentation of *nanite* is available online at https://nanite.readthedocs.io.

## Abbreviations

AFM: Atomic force microscopy
FD: Force-distance
GFP: Green fluorescent protein
MSE: Mean squared error
SVR: Support vector machine regressor
